# Risk decision: The self-charity discrepancies in electrophysiological responses to outcome evaluation

**DOI:** 10.3389/fnhum.2022.965677

**Published:** 2022-10-20

**Authors:** Min Tan, Mei Li, Jin Li, Huie Li, Chang You, Guanfei Zhang, Yiping Zhong

**Affiliations:** ^1^Department of Psychology, Hunan Normal University, Changsha, China; ^2^Cognition and Human Behavior Key Laboratory of Hunan Province, Changsha, China

**Keywords:** self, charity, outcome evaluation, gambling task, event-related potential

## Abstract

Previous studies have examined the outcome evaluation related to the self and other, and recent research has explored the outcome evaluation of the self and other with pro-social implications. However, the evaluation processing of outcomes in the group in need remains unclear. This study has examined the neural mechanisms of evaluative processing by gambling for the self and charity, respectively. At the behavioral level, when participants make decisions for themselves, they made riskier decisions following the gain than loss in small outcomes and engage in more risky behaviors following the loss than gain in large outcomes. However, magnitude and valence did not affect the next risky behavior when participants made decisions for the charity. At the neurophysiological level, the results found that the FRN was larger for the charity outcome than for the self-outcome. For FRN, the valence difference of small outcomes was smaller than that of large outcomes. The P3 response was larger for the self-outcome than for the charity outcome. Meanwhile, compared with the small outcome, the self-charity discrepancies have a significant difference in large outcomes. In addition, the FRN amplitude for self in large outcomes was negatively correlated with the upcoming risky choices, regardless of outcome valence. The behavioral results suggest that people are more likely to optimize strategies for themselves than for the charity. The ERP findings indicated that people focus more on charity outcome than self-outcome in the early stage. In the middle and late stages, people turn attention to their outcomes, and the difference between self’s and charity’s outcome varies with the magnitude. Specifically, it is only in large outcomes that people engage more emotional attention or motivation in their outcomes, but self and charity outcomes had a similar emotional engagement in small outcomes.

## Introduction

It is crucial for people to make decisions quickly according to their behavioral outcomes. The rapid evaluation of outcome is an important cognitive function. Studies have identified FRN and P3 as ERP components related to outcome evaluative processing (Wang et al., 2017; Yang et al., 2018; Liu et al., 2020). For example, FRN and P3 not only represent indicators of early, middle and late processing of outcome evaluation, but also reflect the emotional or motivational level and the allocation of attention resources, respectively (Liu et al., 2020). Among them, FRN is a negative deflection that stems from frontocentral recording sites and peaks 250–300 ms after feedback stimulus (Yeung et al., 2005). The FRN is primarily considered as an indicator of emotional or motivational significance of feedback stimuli or reward prediction errors (Li et al., 2018). Gehring and Willoughby (2002) found that FRN reflects the emotional or motivational significance aroused by the current events. For example, outcomes of the self-executing condition elicited the larger FRN response than those of the observing condition, because of more emotional and motivational relevance in the self-executing condition (Yu and Zhou, 2006). In addition, the FRN is also associated with reward prediction errors, which trigger the greater FRN response when the outcome is worse (vs. better) than expected (Holroyd and Coles, 2002). P3 is a positive component that reaches its peak within 300–600 ms after the feedback stimulus and locates at centroparietal sites (Wu and Zhou, 2009). It is closely related to the allocation of attention resources and the motivational or emotional significance of outcomes (Nieuwenhuis et al., 2005). Self-relevant stimuli preferentially obtain attention resources and have the advantage of processing (Gray et al., 2004; Tacikowski and Nowicka, 2010). In outcome evaluation, self’s outcomes have greater emotional and motivational value and can elicit a larger P3 amplitude than others’ outcomes (Yu and Zhou, 2006).

A large number of studies have explored the neural mechanisms of the self-other decision-making and found that people pay more attention to their than others’ outcomes (Itagaki and Katayama, 2008; Fukushima and Hiraki, 2009; Leng and Zhou, 2010; Tong et al., 2021). Specifically, Yu and Zhou (2006) found that the FRN effect (loss minus gain) elicited by the self-execution condition was larger than the observation condition. Fukushima and Hiraki (2009) further found that one’s own outcomes elicited a greater FRN effect than that elicited by observing competitors’ outcomes. Itagaki and Katayama (2008) further found that the FRN response to one’s own outcomes was greater than that to observing outcomes for partners and competitors. From the perspective of interpersonal relationships, studies found that self-outcomes elicited larger FRN and P3 responses compared to observing friends and strangers (Leng and Zhou, 2010; Tong et al., 2021). Similarly, gambling for oneself elicited a greater brain response than gambling for others, and people allocate more emotional and cognitive resources to their outcomes (He et al., 2018; Liu et al., 2020; Xu, 2021). These results showed obvious the self-other discrepancies.

Prosocial decisions aim to help others in need (Shariff et al., 2016). Decision-making has prosocial implications when the beneficiary is the others or groups in need (Zlatev et al., 2020). However, the other people involved in the previous study did not have prosocial implications, just a stranger not in need or a friend (Itagaki and Katayama, 2008; Fukushima and Hiraki, 2009; Leng and Zhou, 2010; He et al., 2018; Tong et al., 2021). A few studies have explored the outcome evaluation of others with prosocial implications (Liu et al., 2020). However, Liu et al. (2020) only investigated the neural mechanism of outcomes related to a stranger in need, and the outcome evaluation of the group in need is still unclear. Studies have found that people have different psychological and behavioral responses toward single and numerous people who need help (Slovic, 2010). Specifically, people had more emotional experiences with the individual in need and were inclined to help a single individual in need than group in need (Kogut and Ritov, 2005; Small et al., 2007). Given the difference between the individual and group in need, it is necessary to examine self-other decision-making from a group perspective.

Recently, a study explored self-charity discrepancies in risk preference and found that people are more likely to choose the risky option when making decisions for themselves than for charity or a homeless stranger (Zlatev et al., 2020). However, the above study did not explore the neural mechanisms underlying the evaluative processing of one’s own and charity’s outcomes. Additionally, feedback evaluation was influenced by outcome magnitude (Goyer et al., 2008; Gu et al., 2011), but the magnitude effect on the self–other decision-making is also unclear. Therefore, we used ERP techniques to explore the evaluation processing of self and charity. It can help understand the neural mechanism between self and others and enrich the outcome evaluation from the group perspective.

A study found that making decisions for charity was less risky than making decisions for oneself (Zlatev et al., 2020). Therefore, we assumed that the risk rate of making decisions for oneself was significantly higher than that of making decisions for charity. Slovic (2010) found that multiple beneficiaries reduced people’s emotional involvement, we expected that self-elicited larger FRN and P3 responses than charity. Meanwhile, Liu et al. (2020) found that empathic concern only moderated the FRN response and that the valence effect of the FRN was as strong for the stranger outcome in the high-empathy condition (i.e., the stranger-in-need condition) as it was for self-outcome. However, researches have found that people generate relatively less empathy for group in need than the individual in need (Small et al., 2007; Erlandsson et al., 2015). We expected that self-outcome had a larger valence effect on the FRN than the charity outcome. Moreover, compared with small outcomes, large outcomes can bring a larger reward level and have a higher emotional arousal (Xu et al., 2018). We expected outcomes of self and charity to respond differently to different magnitudes.

## Materials and methods

### Participants

A power analysis (G*Power 3.1) suggested that 16 participants would ensure 80% statistical power in the case of small to medium effect sizes (Faul et al., 2007). All participants received 25 yuan for participation and were awarded up to 15 yuan based on their task choice. A total of 35 college students were recruited from Hunan Normal University, and four of them were excluded from the subsequent analysis due to a lack of valid trials for a certain condition, including 31 valid subjects (*M*_*age*_ = 19.45, SD = 1.20, 16 female). All participants had no history of neurological or psychiatric disorders. They had normal or corrected vision and were right-handed.

### Procedure

**FIGURE 1:**
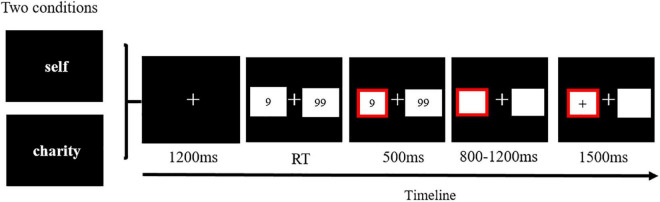
An illustration of a single trial.

Participants were required to perform a gambling task for themselves and charity (China Charity Federation). The task was to choose between the numbers 9 and 99, and the credits they selected could be gained or lost according to the feedback after making the choice (Yang et al., 2018). The credits for the same beneficiary kept accumulating during the task. The credits accumulated in the task can be converted into cash in a certain proportion and determined participants’ and the charity’s reward. Among them, we made real and anonymous donations to charity. The whole experiment included a practice and a formal experiment. The practice experiment included 16 trials. Only after the participants completely understood the task rules did they start the formal experiment. The formal task consists of two blocks (480 trials), 240 trials each. However, what the participants did not know was that the probability of winning or losing feedback was 0.5. That is, a block had 120 positive and negative feedback in the experiment setting. However, because each participant had the different number of small and large outcomes, we could only achieve a similar total number of positive and negative feedback in each participant’s choice number of the large and small outcomes. The average number of trials for each condition was shown in Table 1. Each block began with a cue (for self and charity). Each trial started from the central fixation point of 1200 ms, and then the participants were asked to press F or J on the keyboard to choose between 9 and 99. Then, the selected option was highlighted in red and displayed for 500 ms. Thereafter, blank rectangles were randomly presented at 800–1200 ms on the screen. Positive or negative feedback was presented in the rectangle of the selected number (see Figure 1).

**TABLE 1:** The mean trials and discarded trials of all the experimental conditions.

Conditions		Gain	Loss
Charity	Small outcome	63.32 (5.06)	61.39 (4.84)
	Large outcome	57.23 (4.91)	58.06 (4.32)
Self	Small outcome	67.39 (3.74)	67.45 (3.26)
	Large outcome	52.81 (2.55)	52.35 (2.03)

The value in parentheses is the mean of discarded trials in the EEG analysis.

### EEG recording and analysis

We used 32 scalp sites of Brain Products to record the electrical activity of the brain. A horizontal electrooculogram (EOG) was recorded by placing electrodes above both eyes. Meanwhile, the sampling rate was 500 Hz/channel, and the filter bandpass was 0.05–100 Hz. The impedance between all electrodes and scalp was less than 5 kΩ. The online reference electrode is Fz, and the offline reference is the average of the left and right mastoids. After the continuous recording of EEG data, offline analysis was performed. EEG data were analyzed using the EEGLAB toolkit. First, the data are filtered with parameters from 0.1 to 30 Hz (filter slopes: 24 dB/octave). Then, ICA was used to remove EOG and artifacts. Then, the data was segmented for a period from 200 ms before feedback onset to 1000 ms. Finally, we excluded artifacts with wave amplitude greater than ±100 μV. Combined with a visual inspection of brain topography and waveform and based on the previous literature and experimental purposes (Hu et al., 2017; Yang et al., 2018; Liu et al., 2020), we calculated the mean values of FRN amplitude within 220–310 ms window after the outcome feedbacks were presented and used the mean value within 310–420 ms time window to calculate P3. F3, Fz, F4, FC1, FC2, C3, Cz, and C4 were selected as the analytical electrodes of FRN, while C3, Cz, C4, CP1, CP2, Pz, P3, and P4 were selected as the analytical electrodes of P3. After data processing, we determined the average trials under each condition (“charity-small gain”: 58.26, “charity-small loss”: 56.55, “charity-large gain”: 52.32, “charity-large loss: 53.74, “self-small gain”: 63.65, “self-small loss”: 64.19, “self-large gain”: 50.26, “self-large loss”: 50.32, *F*(7, 240) = 1.77, *p* = 0.094). We performed the average amplitude of FRN and P3 on 2 (beneficiary: self vs. charity) × 2 (valence: gain vs. negative) × 2 (magnitude: large vs. small) repeated-measures analysis of variance (ANOVA). All data analyses were performed using SPSS 26.

## Results

### Behavior results

We define 99 as the risky option (Yang et al., 2018). The paired sample *t*-test was used to compare the ratio of risky choices among the self and charity, but there was not significant difference [*t*(30) = 1.30, *p* = 0.204]. We further examined the effect of feedback on the next risk-taking behavior, and the ratio of 99 on the next trial was chosen by the participants as the dependent variable. We conducted a 2 (beneficiary: self vs. charity) × 2 (valence: gain vs. loss) × 2 (magnitude: large vs. small) repeated-measures ANOVA. The main effect of the magnitude was significant [*F*(1,30) = 19.77, *p* < 0.001, $\eta_{p}^{2}$ = 0.40]. Consistent with previous studies (Yang et al., 2018), participants chose more high-risk options following a large outcome than a small outcome (54.1 ± 19.0% vs. 37.8 ± 17.7%). The interaction between magnitude and valence was significant [*F*(1,30) = 8.96, *p* = 0.005, $\eta_{p}^{2}$ = 0.23]. More importantly, the effect was moderated by beneficiary [*F*(1,30) = 9.42, *p* = 0.005, $\eta_{p}^{2}$ = 0.24]. When the beneficiary was oneself, the interaction between magnitude and valence was significant [*F*(1,30) = 25.95, *p* < 0.001, $\eta_{p}^{2}$ = 0.46]. This simple effect found that participants were more likely to choose high-risk options following small positive outcomes than small negative outcomes (40.7 ± 20.6% vs. 32.2 ± 18.6%), but participants chose fewer high-risk options following large positive outcomes than large negative outcomes (48.8 ± 20.8% vs. 58.0 ± 18.4%). The interaction between magnitude and valence was not significant when the beneficiary was the charity [*F*(1,30) = 0.10, *p* = 0.756]. Other effects were not significant [*F*s(1,30) < 1.19, *p*s > 0.29].

### The FRN results

**FIGURE 2:**
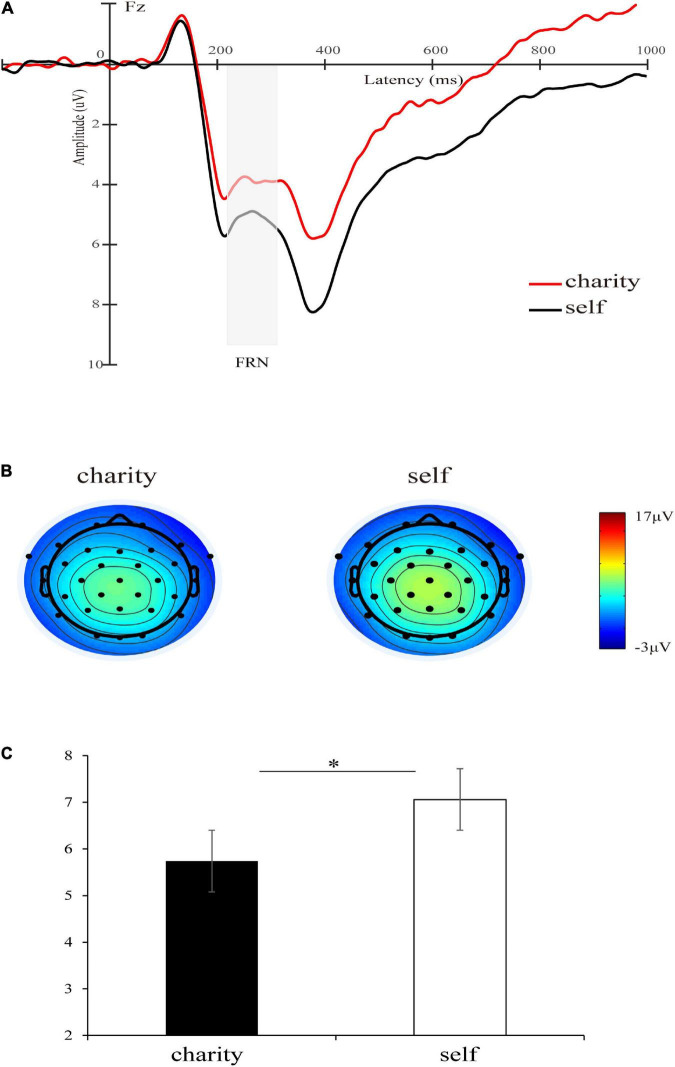
**(A)** Grand-average ERP waveforms of FRN at Fz electrode site, the gray area highlights the 220–310 ms time window for calculating the mean value of the FRN amplitude. The red line represents the waveform of the charity and the black represents the waveform of self. **(B)** Topographic maps of charity and self-condition. **(C)** The bar graphs and standard errors show the mean values of FRN for charity and self-condition. *p < 0.05.

**FIGURE 3:**
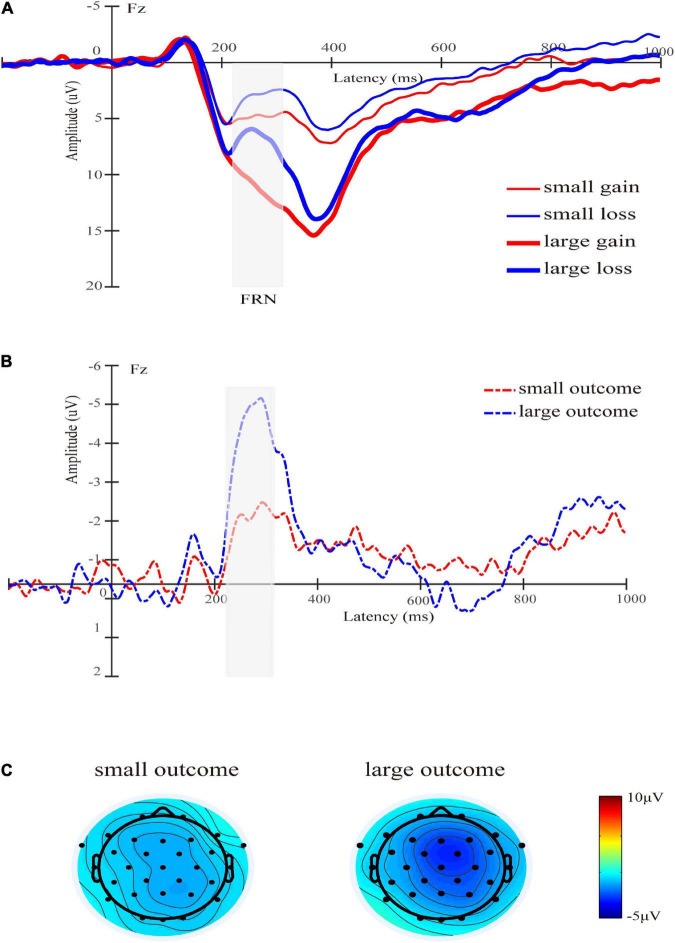
**(A)** Grand-average ERP waveforms of FRN at Fz electrode site. The gray area highlights the 220–310 ms time window for calculating the mean value of FRN. The thin red line represents the waveform with a small gain condition, the thin blue line represents the waveform with a small loss condition, the thick red line represents the waveform with a large gain condition, and the thick blue line represents the waveform with a large loss condition. **(B)** The difference wave of FRN in the 220–310 ms time window (loss minus gain). **(C)** The scalp topographies of the difference for small and large outcomes are presented.

**FIGURE 4:**
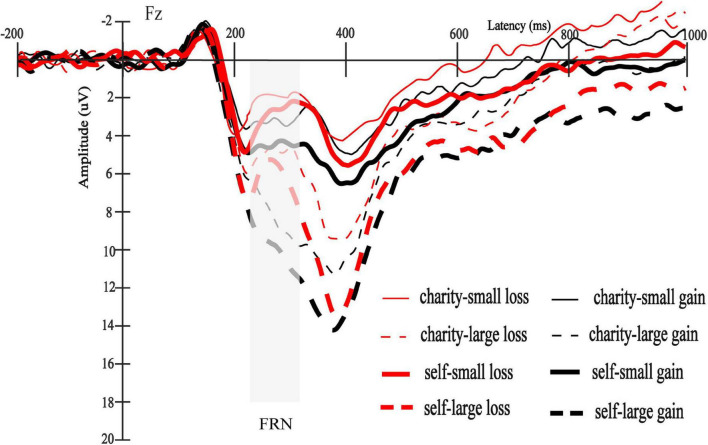
The total average FRN waveforms for all conditions.

The main effect of the beneficiary was significant [*F*(1,30) = 7.05, *p* = 0.013, $\eta_{p}^{2}$ = 0.19], and the FRN elicited by the charity was larger than self (5.74 ± 3.54 μV vs. 7.06 ± 3.94 μV) (see Figure 2). The main effect of the magnitude was significant [*F*(1,30) = 57.92, *p* < 0.001, $\eta_{p}^{2}$ = 0.66], and the FRN elicited by small outcomes was larger than that elicited by large outcomes (4.48 ± 3.44 μV vs. 8.33 ± 4.05 μV), which was consistent with the previous studies (Gu et al., 2011). Also consistent with the previous findings (Yang et al., 2018), the main effect of valence was significant [*F*(1,30) = 36.68, *p* < 0.001, $\eta_{p}^{2}$ = 0.55], and the loss outcomes elicited a larger FRN than the gain outcomes (5.33 ± 2.99 μV vs. 7.48 ± 4.15 μV). The interaction between magnitude and valence was significant [*F*(1,30) = 8.15, *p* = 0.008, $\eta_{p}^{2}$ = 0.21], and the valence difference in the small outcomes [5.16 ± 3.99 μV vs. 3.80 ± 3.24 μV, *F*(1,30) = 10.24, *p* = 0.003,$\eta_{p}^{2}$ = 0.25] was smaller than the valence difference of large outcomes [9.80 ± 4.84 μV vs. 6.86 ± 3.59 μV, *F*(1,30) = 37.90, *p* < 0.001,$\eta_{p}^{2}$ = 0.56]. To explore the interaction further, we analyzed the mean amplitude of the FRN on the difference waves (loss minus gain). We found that the FRN effect (loss minus gain) of large outcomes was larger than that of small outcomes [−2.94 ± 2.66 μV vs. −1.35 ± 2.35 μV, *t* (30) = 2.86, *p* = 0.008, d = 1.04] (see Figure 3). Other interactions were not significant [*F*s(1,30) < 1.62, *p*s > 0.21]. The total mean waveforms for all conditions were shown in Figure 4.

### The P3 results

The main effect of the beneficiary was significant [*F*(1,30) = 10.43, *p* = 0.003, $\eta_{p}^{2}$ = 0.26], and P3 amplitude elicited by self was larger than charity (11.29 ± 5.64 μV vs. 8.78 ± 4.34 μV). The main effect of magnitude was significant [*F*(1,30) = 56.31, *p* < 0.001,$\eta_{p}^{2}$ = 0.65], and the P3 elicited by large outcomes was larger than that elicited by small outcomes (13.03 ± 5.85 μV vs. 7.04 ± 4.11 μV), which was consistent with the previous studies (Liu et al., 2020). Consistent with the previous findings (Kou et al., 2022), the main effect of valence was significant [*F*(1,30) = 22.19, *p* < 0.001, $\eta_{p}^{2}$ = 0.43], and gain outcomes elicited larger P3 amplitude (10.70 ± 4.86 μV vs. 9.37 ± 4.34 μV) than the loss outcomes.

**FIGURE 5:**
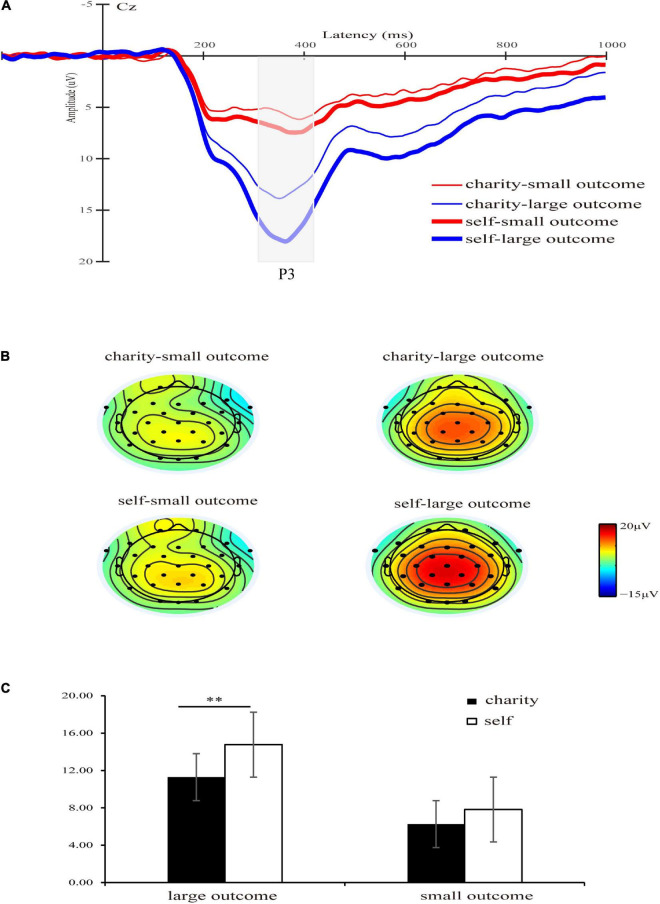
**(A)** Grand-average ERP waveforms of P3 at Cz electrode site, the gray area highlights the 310–420 ms time window for calculating the mean value of the P3 amplitude. The thin red line represents the waveform with a charity-small outcome condition, the thin blue line represents the waveform with a charity-large outcome condition, the thick red line represents the waveform with a self-small outcome condition, and the thick blue line represents the waveform with a self-large outcome condition. **(B)** Topographic maps of P3 of each condition. **(C)** The bar graphs and standard errors show the mean values of P3 for each condition. **p < 0.01.

**FIGURE 6:**
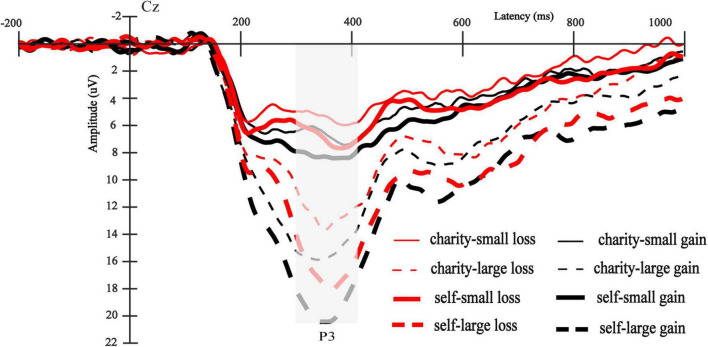
The total average P3 waveforms for all conditions.

The interaction between beneficiary and magnitude was significant [*F*(1,30) = 6.32, *p* = 0.018, $\eta_{p}^{2}$ = 0.17]. There was no significant difference between self and charity in small outcomes [7.82 ± 4.76 μV vs. 6.26 ± 4.59 μV, *F*(1,30) = 3.82, *p* = 0.060, $\eta_{p}^{2}$ = 0.11], but the difference between self and charity was significant in large outcomes [14.76 ± 7.04 μV vs. 11.29 ± 5.68 μV, *F*(1,30) = 13.99, *p* = 0.001, $\eta_{p}^{2}$ = 0.32] (see Figure 5). Other interactions were not significant [*F*s (1,30) < 1, *p*s > 0.46]. The total average P3 waveforms for all conditions were shown in Figure 6.

### Correlation analysis

Some studies have shown that FRN is related to the risky decision of the next trial (Hewig et al., 2007; Kiat et al., 2016), and the interaction between magnitude and valence has also been found on the FRN amplitude, which is consistent with the behavioral results. Therefore, we conducted the correlation analysis between FRN and behavior. The separate correlation analyses were conducted between magnitude and valence according to the different beneficiaries. This result found that FRN amplitudes were significantly negatively correlated with both positive and negative large outcomes involving the self (*r_*gain*_* = −0.50, *p* = 0.004, *r_*loss*_* = −0.39, *p* = 0.032), but this pattern of correlation was absent in small outcomes (*r*_*gai*n_ = 0.04, *p* = 0.833, *r_*loss*_* = −0.28, *p* = 0.122). Meanwhile, we also found a related trend between FRN amplitudes and large positive outcomes involving the charity (*r*_*gai*n_ = −0.32, *p* = 0.082). The correlation analysis results were shown in Table 2.

**TABLE 2:** Correlation analysis results.

Conditions		*r*	*p*
Charity	+9	–0.11	0.543
	−9	0.13	0.503
	+99	–0.32	0.082
	−99	–0.15	0.434
Self	+9	0.04	0.833
	−9	–0.28	0.122
	+99	−0.50**	0.004
	−99	−0.39*	0.032

Correlation coefficient is statistically significant at *p < 0.05, **p < 0.01.

## Discussion

This study examined outcome evaluation related to self and charity. This result showed that self and charity have similar risk-taking behaviors. Our findings indicate that the outcomes of the charity and self are different at two stages of evaluation. The results are discussed in detail by risky ratio, FRN and P3, respectively.

The results showed that the risky behavior between self and charity was similar, which is inconsistent with the hypothesis. Zlatev et al. (2020) found that people were more likely to take risks when making decisions for themselves than for charity or stranger-in-need. In contrast, Liu et al. (2020) found that the stranger-in-need and self were similar in risky behaviors involving outcome evaluation. This may be caused by differences in the operational definition of risky behavior. The risky option was a larger reward with a probability of 75% and $0 with a probability of 25%, while the non-risky option was a small, certain reward (Zlatev et al., 2020). However, both large and small rewards had a 50% chance of loss or gain in the outcome evaluations (Liu et al., 2020), which were consistent with our study. Studies have shown that probability affects risk-taking behavior (Sun et al., 2009). Therefore, the probability of risky behaviors in studies may cause inconsistency in results. In addition, at the behavioral level, when participants made decisions for themselves, they are more likely to seek risk to maximize their self-interest after positive feedback than negative feedback in the small outcome. However, they were more likely to choose the high-risk option after high-risk choice with negative feedback in the large outcome, which is consistent with previous studies (Schuermann et al., 2012). High-risk behavior may be intended to avoid negative consequences in the future, since conservative behavior after the large loss cannot compensate for the loss. And people are more willing to protect the money they have had and to act more conservatively after the large gain. When participants made decisions for the charity, this risk-taking behavior was unaffected by the magnitude and valence of the feedback, i.e., the same strategy was used for both large and small outcomes. This suggests that people are more likely to optimize decisions for their own interests than for the charity’s interests.

FRN reflects emotional or motivational evaluation of the current outcome at an early stage (Gehring and Willoughby, 2002). We found that charity outcomes elicited greater FRN responses than self-outcomes. This was inconsistent with our hypothesis that people had stronger emotional concern toward single rather than multiple beneficiaries (Slovic, 2010). This may be because, compared with individualism, collectivism is more about collective than individual interests (Chen et al., 1997). Chinese participants recruited by this experiment would pay more emotional attention to the collective outcomes. Another possible explanation is that this represents a difference in expected errors. According to the reinforcement learning-error-related negativity theory, FRN is sensitive to expectancy violations (Holroyd and Coles, 2002; Hewig et al., 2011). In other word, this causes a greater FRN response when the actual outcomes do not match the expected outcomes. The homo economicus assumption holds that humans are self-interested actors (Smith, 1776). People are more likely to expect their own outcomes from this assumption. Thus, this gap between actual outcomes and previous expectations triggered a larger FRN response when charity outcomes were presented.

The results found that loss elicited a larger FRN response than gain for large outcomes, while this effect was weaker for small outcomes. This was consistent with previous studies (Yang et al., 2018). Some studies have shown that the FRN showed a relatively positive deflection in the reward condition (San Martín et al., 2010). Positive emotions induce activities of the midbrain dopamine system, which makes the brain more sensitive to rewards, leading to the positive deflection for FRN (Mushtaq et al., 2016). This suggests that gain induces a smaller FRN response than loss. The magnitude of the outcome is the index of the level of emotion or motivation and has different benefit levels (San Martín, 2012). The reduction of the reward level also decreases people’s emotions and motivation levels. Therefore, the FRN effect for large outcomes is larger than the FRN effect for small outcomes. This suggests that FRN can recognize the rank of outcomes, and large outcomes carry greater weight in emotional and motivational relevance.

However, the results found that the FRN effect of charity was as strong as that of self, which was inconsistent with the hypothesis. The greater the number of people in need, the less empathy generates (Erlandsson et al., 2015). In other words, charity can evoke less empathy than an individual in need, which can lead to differences in emotional salience between the self and charity. But Chinese people pay more attention to group interests generally (Chen et al., 1997), which may compensate for this difference between self and charity. Therefore, the FRN effects of charity and self were similar. There is another possible explanation. From the perspective of expectancy violation, some studies have found that people always expect the outcome of helping others to be successful, so the failed feedback induces a stronger FRN than the successful feedback when helping others (Gan et al., 2016, 2022). In the same way, people also expect to get better self-outcomes to maximize the self-interest, so loss also induces a greater FRN response than gain. In short, people have the same expectations of themselves and charity outcomes, so the FRN effects of charity and self are similar.

Moreover, charity and self-outcomes were not affected by the magnitude in the FRN amplitude. This is not consistent with the hypothesis. A possible reason is that the participants’ concern for group interests reduced the discrepancy between the charity and themselves in different magnitudes. Another factor might be that the charity is pro-social. Studies have shown that prosocial behaviors can experience vicarious rewards, such as happiness (Dunn et al., 2008; Aknin et al., 2013). Specifically, prosocial behavior refers to the involvement of the ventromedial prefrontal cortex (VMPFC) (Morelli et al., 2015), which monitors the subjective value of behavioral outcomes and guides decision-making by increasing the motivational significance of behaviors (Carlson et al., 2016). These results indicate that charitable outcomes will activate people’s higher motivational relevance and emotion levels. Therefore, for both large and small outcomes, the FRN response of charitable outcomes was greater than that of self-outcomes. In other words, this prosocial involvement weakens hierarchically magnitude sensitivity in the early stage. Interestingly, the FRN amplitude for self in large outcomes was negatively associated with possibility of making risky decisions on the next trial, while this correlation was absent in small outcomes. High-risk decisions may also reflect the motivational or emotional significance compared with low-risk decision (Schuermann et al., 2012), and larger outcomes also activate greater levels of emotion or motivation (San Martín, 2012). Thus, the FRN amplitude was significantly negatively correlated with large outcomes for the self. However, small outcomes reduce people’s motivational or emotional level, which weakens the correlation between FRN amplitude and future risky behaviors.

P3 reflects the significance of emotion or motivation, which can process current events in a relatively accurate manner (Nieuwenhuis et al., 2005). It was found that self-outcomes evoked larger P3 amplitude than charity outcomes, which was consistent with the previous studies that self-interest evoked greater motivational or emotional significance (Yu and Zhou, 2006). This suggests that, in the middle and later stages, participants focus more on self-outcomes and allocate more attention resources to themselves. Importantly, we found that self-elicited larger P3 amplitude than charity for large outcomes. However, with small outcomes, the difference disappears. The motivational significance can be measured by the magnitude of the outcomes (San Martín, 2012). Specifically, the magnitude of the outcomes represents different levels of reward, and large outcomes activate greater interests and have stronger motivational significance and emotional response than the small ones. Self-gains and losses in large outcomes are associated with higher levels of self-interest, evoking higher levels of emotion and motivation. Consequently, this increases the level of emotion and motivation for self-outcomes. However, small outcomes reduce the level of reward and people’s arousal to self-interest, which makes people have a similar P3 amplitude for self and charity in small outcomes.

Our study has several limitations. First, our risk behavior mainly involves binary gambling tasks. However, in everyday life, we face situations more complex than simple binary game tasks. Meanwhile, this paradigm lacks real-life scenarios, which may make it difficult to generalize the experimental results to real life. So, future research could explore differences in outcome evaluation by asking participants to perform more complex decision tasks or more realistic tasks for themselves and charity. Moreover, our sample comes from China with a collectivist culture. Compared with Chinese participants who focus on collective interests, participants from western cultures may show different outcome processing. Therefore, future cross-cultural research needs to confirm this difference in outcome evaluation between self and charity. Although we have attributed the self-charity discrepancies in electrophysiological responses to emotional or motivational salience, expectancy violations as mentioned in the discussion may also play a role. Particularly, P3 is related to action updating (Donchin and Coles, 1988; Yang et al., 2018). The action updating may also has a potential impact in outcome evaluation. Future research could examine the effect of other factors, such as action updating, given that P3 has various cognitive functions. Finally, we did not directly compare individuals in need with charity and could not account for the difference in outcome evaluation between individuals and groups with prosocial implications. Future research can explore the difference between individual and group levels.

## Conclusion

This study has used ERP technology to explore differences in outcome evaluation between self and charity. People are more likely to adjust strategies for their own outcomes than for charity outcomes from behavioral results. Meanwhile, in the early stage, individuals paid more emotional investment to charity than to their outcomes (FRN as the indicator). In the middle and later stages, individuals focus more on their outcomes than on the outcomes of the charity (P3 as the indicator). Moreover, the difference in emotional or motivational concerns between self and charity was only moderated by the magnitude in the middle and late stages. In other words, individuals focus more on their outcomes than on the outcomes of the charity in large outcomes, while self and charity have similar emotional or motivational concerns for small outcomes.

## Data availability statement

The raw data supporting the conclusions of this article will be made available by the authors, without undue reservation.

## Ethics statement

The studies involving human participants were reviewed and approved by the Research Ethics Board of Hunan Normal University. The patients/participants provided their written informed consent to participate in this study.

## Author contributions

MT, ML, and YZ designed the experiment. MT, HL, and CY carried out the experiments. MT, ML, and JL analyzed the data. MT and GZ wrote the manuscript. All authors edited and approved the manuscript.
